# The association between inflammatory potential of diet and disease activity: results from a cross-sectional study in patients with inflammatory bowel disease

**DOI:** 10.1186/s12876-020-01435-4

**Published:** 2020-09-29

**Authors:** Carlijn R. Lamers, Nicole M. de Roos, Ben J. M. Witteman

**Affiliations:** 1 Division of Human Nutrition and Health, Wageningen University & Research (WUR), Stippeneng 4, 6708 WE Wageningen, The Netherlands; 2 Department of Gastroenterology and Hepatology, Hospital Gelderse Vallei, Ede, The Netherlands

**Keywords:** Crohn’s disease, Ulcerative colitis, Diet, Inflammation, Dietary inflammatory index

## Abstract

**Background:**

Diet may play a role in disease status in patients with inflammatory bowel disease. We tested whether the inflammatory potential of diet, based on a summation of pro- and anti-inflammatory nutrients, is associated with disease activity in patients with Crohn’s disease and ulcerative colitis.

**Methods:**

Participants completed a disease activity questionnaire (short Crohn’s Disease Activity (sCDAI) or Patient Simple Clinical Colitis Activity Index (P-SCCAI)) and a Food Frequency Questionnaire (FFQ). FFQ data were used to calculate the Dietary Inflammatory Index (DII) which enables categorization of individuals’ diets according to their inflammatory potential on a continuum from pro- to anti-inflammatory. Associations with disease activity were investigated by multiple linear regression.

**Results:**

The analysis included 329 participants; 168 with Crohn’s disease (median sCDAI score 93 [IQR 47–156]), and 161 with ulcerative colitis (median P-SCCAI score 1 [IQR 1–3]). Mean DII was 0.71 ± 1.33, suggesting a slightly pro-inflammatory diet. In Crohn’s disease, the DII was positively associated with disease activity, even after adjustment for confounders (*p* = 0.008). The mean DII was significantly different between participants in remission and with mild and moderately active disease (0.64, 0.97 and 1.52 respectively, *p* = 0.027). In ulcerative colitis, the association was not significant.

**Conclusions:**

Disease activity was higher in IBD participants with a more pro-inflammatory diet with statistical significance in Crohn’s disease. Although the direction of causality is not clear, this association strengthens the role for diet in medical treatment, which should be tested in an intervention study.

## Background

Dietary intake seems to play a role in the development of Crohn’s disease (CD) and ulcerative colitis (UC), and possibly also in maintenance of remission and improvement of quality of life in patients with one of these inflammatory bowel diseases (IBD) [1, 2]. The exact mechanism is unknown, but modification of the gut microbiota and influence on immunological processes seem to be important [3, 4].

Diet is a modifiable lifestyle factor in which IBD patients seem to be interested and that may have beneficial effects on the course of IBD. Surveys performed on dietary beliefs and behaviour showed that around 60% of patients believe that diet influences their disease course and up to 77% of patients reported avoidance of particular foods to prevent or treat a flare [5–7]. There is cumulative evidence that certain components of a diet have anti- or pro-inflammatory properties and may therefore influence the course of disease [3, 8]. This has led to the development of the Dietary Inflammatory Index (DII). With this DII, diets can be categorized from maximally anti-inflammatory to maximally pro-inflammatory. Assessment of the inflammatory potential of a diet takes into account that some foods contain both beneficial and unhealthy nutrients. Therefore, it is likely that the DII better reflects the influence of diet on inflammation and thus on the course of disease than analyses with single foods [9–11]. The DII has been used in several other patient groups to predict the dietary inflammatory potential related risk for development of disease or the influence on clinical course of disease [12–14].

Therefore, the aim of this study was to assess the association between the inflammatory potential of diet and disease activity in patients with inflammatory bowel disease, separated into CD and UC patients. We also investigated participants’ self-perceived impact of diet on disease and whether they had made dietary modifications.

## Methods

### Study design and study population

In this cross-sectional study, IBD patients aged 18 years or older, diagnosed with either CD or UC, were included. Participants were recruited between July and October 2018. A total of 1035 patients received a personal invitation: 940 IBD patients of a local regional hospital in the Netherlands received a letter and 95 IBD patients from the Nijmegen Exercise Study - a longitudinal study to examine the impact of a physically active lifestyle on health, quality of life, development and progression of various (chronic) diseases - received an email with an invitation to participate. Furthermore, an unknown number of patients responded to an invitation via digital newsletters and the website of the Dutch IBD patient association. In total, 397 patients sent us an e-mail to show interest in the study and received access to our online questionnaire. Unfortunately, we did not register the source each participant originated from. Participation comprised an online questionnaire composed of questions regarding participants characteristics, a disease activity questionnaire (short Crohn’s Disease Activity (sCDAI) or Patient Simple Clinical Colitis Activity Index (P-SCCAI)) and a Food Frequency Questionnaire (FFQ) [15–17]. It took participants about 40 min to complete the whole questionnaire. Participants were excluded in case of indeterminate colitis or unknown IBD type, missing or incomplete FFQ data or an implausible energy intake (< 800 or > 4000 kcal per day for men and < 500 or > 3500 kcal per day for women) to limit errors due to misreporting [18].

The medical ethical committee of Wageningen University decided that no formal ethical approval was needed, due to the low burden and risk of the study. All participants provided digital informed consent.

### Data collection

#### Participant characteristics

Information on age, gender, height and weight, level of education, type of IBD, age at diagnosis, current medication and supplement use, previous IBD-related surgeries, food allergies and smoking was retrieved from the online questionnaire.

#### Disease activity

Disease activity was evaluated using the short Crohn’s Disease Activity Index (sCDAI) for CD and the Patient Simple Clinical Colitis Activity Index (P-SCCAI) for UC [15, 16]. Disease activity scores were used as a continuous outcome measure and classified using previously validated cut-off points: remission (sCDAI < 150 or P-SCCAI ≤2), mildly active disease (sCDAI 150–219 or P-SCCAI 3–5), moderately active disease (sCDAI 220–450 or P-SCCAI 6–11) and severely active disease (sCDAI > 450 or P-SCCAI ≥12) [15, 19].

#### Dietary intake

Dietary intake was assessed using a 179-item validated Food Frequency Questionnaire (FFQ) designed to assess the intake of the Dutch population by capturing the foods consumed during the previous month [17]. Macronutrient intake was calculated and the FFQ was used to calculate the inflammatory potential of their habitual diet by using the DII. The DII is an index consisting of 45 food parameters developed by reviewing and scoring scientific articles on diet and inflammatory markers to be able to determine the inflammatory potential of a diet [9, 10]. A DII score above zero represents a pro-inflammatory diet and a DII score below zero represents an anti-inflammatory diet. Several papers described the development of inflammatory indices and calculation of the DII [9–11]. In short, DII scores of each food parameter were calculated by subtraction of the standard global mean of a representative world database from the amount of the food parameter eaten estimated from an FFQ and dividing this value by its standard deviation [10]. This Z-value was converted to a centred percentile score to minimize the effect of skewing and to achieve a symmetrical distribution. This centred percentile score was multiplied by the food parameter specific inflammatory effect score. All food parameter DII scores were summed to create an overall DII score. When calculated from all 45 parameters, the DII could theoretically range from − 8.87 (maximally anti-inflammatory) to + 7.98 (maximally pro-inflammatory) [20]. In our study, data on 28 of the 45 parameters were available for inclusion in the overall DII score, namely: energy, protein, carbohydrate, total fat, saturated fat, cholesterol, trans fat, mono- and polyunsaturated fat, n-3 and n-6 fatty acids, fibre, thiamine, riboflavin, niacin, vitamins A, B6, B12, C, D and E, zinc, iron, magnesium, selenium, folic acid, beta-carotene and alcohol. The remaining 17 parameters, mainly flavonoids, herbs and spices, were not available because not all food parameters could be assessed reliably with the FFQ we used or were not available in the food composition database.

#### Patient reported impact and modification of diet

Information on participants’ self-perceived impact of diet on disease and whether they had made dietary modifications since their diagnosis was retrieved from the online questionnaire.

### Statistical analysis

Normally distributed data are presented as mean ± standard deviation (SD), skewed data as median with interquartile range (IQR) and categorical data as frequencies with proportions. To compare baseline characteristics and DII between CD and UC and between disease activity groups, Chi-square tests were performed for categorical data, and independent samples t-test and one-way analysis of variance (ANOVA) (or Kruskall-Wallis when not normally distributed) were performed for continuous variables. Post-hoc analyses for disease activity groups were performed using the Bonferroni multiple comparisons test. Multiple linear regression was used to determine associations between inflammatory potential of diet and disease activity. Results were reported as β-coefficients with 95% confidence intervals (CI). A *p*-value of < 0.05 was considered statistically significant. Statistical analysis was carried out using IBM SPSS Statistics version 24.

## Results

### Participants characteristics

**Fig. 1:**
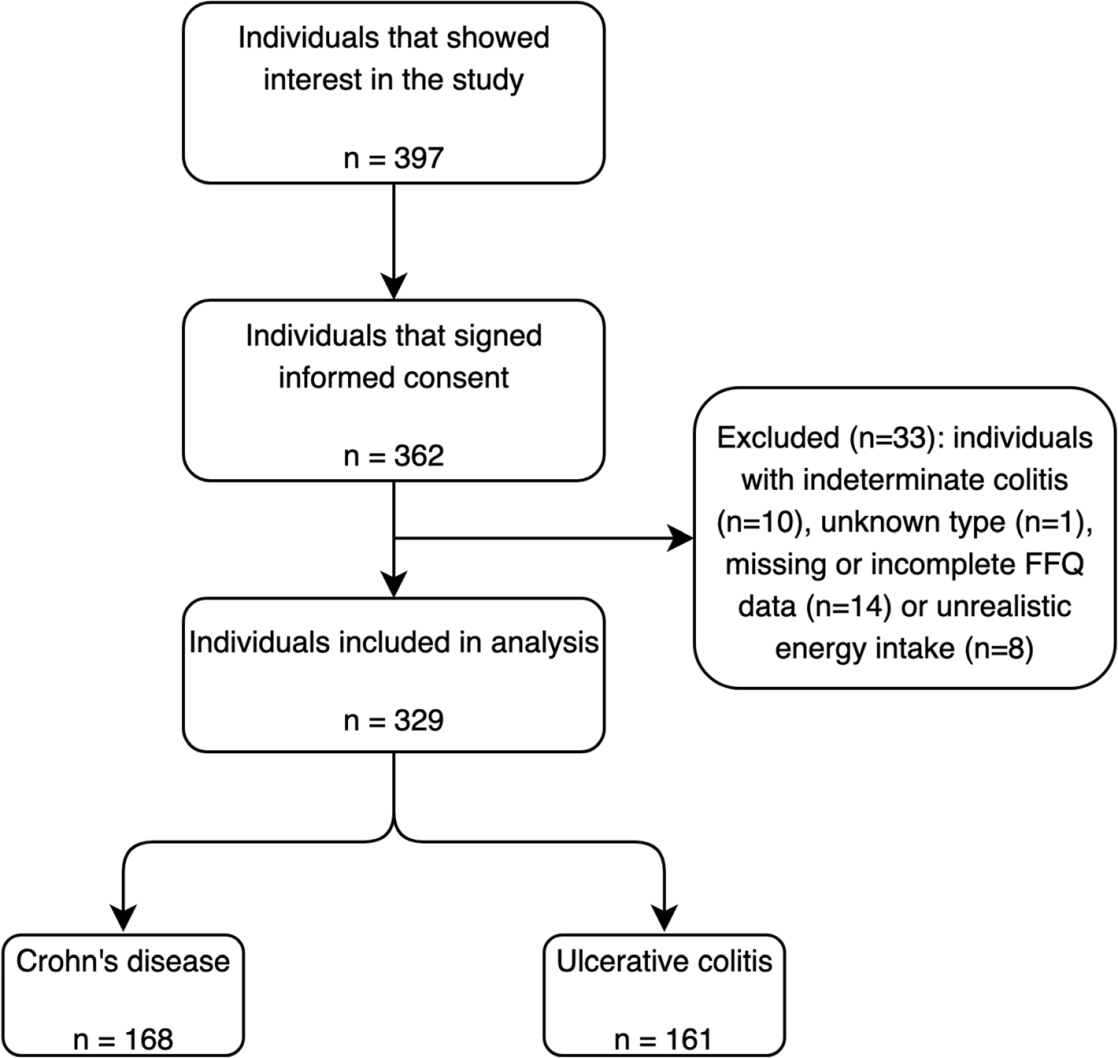
Flowchart of individuals included in analysis

In total, 329 participants were included in the analysis (Fig. 1). Of these 329 participants, 168 participants (51%) had CD and 161 participants (49%) had UC. The majority of participants was female, well-educated and had experienced two or less flare-ups in the last year. The number of participants classified as having severely active disease was too small to analyse as a separate group (UC, *n* = 2), so they were included in the moderately active disease group. About two-thirds of participants were in remission. Participants with CD were slightly younger at the time of this study and at diagnosis, they used more immunosuppressants and biologicals, and had more IBD-related surgeries than patients with UC. Supplement use was comparable in CD and UC (Table 1).

**Table 1:** Characteristics of the study population consisting of 168 CD and 161 UC participants

			Disease activity CD			Disease activity UC		
	CD	UC	Remission	Mild	Moderate	Remission	Mild	Moderate
Subjects, n (%)	168 (51)	161 (49)	123 (73)	27 (16)	18 (11)	108 (67)	38 (24)	15 (9)
Gender, n (%)								
Female	117 (70)	97 (60)	84 (68)	17 (63)	16 (89)	60 (56)	27 (71)	10 (67)
Age (years)	47 ± 16*	51 ± 15*	48 ± 16	46 ± 17	47 ± 14	50 ± 14	54 ± 16	45 ± 16
Age at diagnosis (years)	32 ± 15*	36 ± 15*	33 ± 16	30 ± 14	33 ± 9	35 ± 14	41 ± 17	32 ± 15
BMI (kg/m^2^)	24.8 ± 4.7	24.9 ± 3.8	24.8 ± 4.4	24.9 ± 5.1	25.0 ± 6.6	24.7 ± 3.6	25.4 ± 3.9	24.8 ± 4.9
Smoking, n (%)								
Never	123 (73)	124 (77)	97 (79)^a^	17 (63)^a, b^	9 (50)^b^	80 (74)	31 (82)	13 (87)
Current	16 (10)	7 (4)	8 (7)^a^	6 (22)^b^	2 (11)^a, b^	5 (5)	1 (3)	1 (7)
Former	29 (17)	30 (19)	18 (15)^a^	4 (15)^a, b^	7 (39)^b^	23 (21)	6 (16)	1 (7)
Education level^c^, n (%)								
Low	37 (22)	34 (21)	25 (20)	7 (26)	5 (28)	21 (19)	10 (26)	3 (20)
Middle	52 (31)	48 (30)	38 (31)	5 (19)	9 (50)	33 (31)	8 (21)	7 (47)
High	79 (47)	79 (49)	60 (49)	15 (56)	4 (22)	54 (50)	20 (53)	5 (33)
Medication use, n (%)								
Mesalazines	28 (17)**	97 (60)**	21 (17)	4 (15)	3 (17)	65 (60)	21 (55)	11 (73)
Corticosteroids	22 (13)	24 (15)	13 (11)	6 (22)	3 (17)	9 (8)^a^	10 (26)^b^	5 (33)^b^
Immunosuppressants	70 (42)**	33 (21)**	52 (42)	11 (41)	7 (39)	20 (19)	9 (24)	4 (27)
Biologicals	58 (35)**	27 (17)**	36 (29)	12 (44)	10 (56)	11 (10)^a^	9 (24)^a, b^	7 (47)^b^
Other	24 (14)*	11 (7)*	14 (11)	6 (22)	4 (22)	4 (4)^a^	4 (11)^a, b^	3 (20)^b^
No medication use	34 (20)	30 (19)	29 (24)	4 (15)	1 (6)	24 (22)	6 (16)	0 (0)
Flare-ups in past year, n (%)								
None	92 (55)*	61 (38)*	80 (65)^a^	7 (26)^b^	5 (28)^b^	50 (46)^a^	10 (26)^a, b^	1 (7)^b^
1–2 flare-ups	55 (33)*	70 (44)*	35 (29)	14 (52)	6 (33)	45 (42)	20 (53)	5 (33)
3–4 flare-ups	8 (5)*	19 (12)*	2 (2)^a^	4 (15)^b^	2 (11)^a, b^	8 (7)^a^	4 (11)^a^	7 (47)^b^
More than 4 flare-ups	13 (7)	11 (7)	6 (5)^a^	2 (7)^a, b^	5 (28)^b^	5 (5)	4 (11)	2 (13)
Supplement use, n (%)	74 (44)	68 (42)	54 (44)	11 (41)	9 (50)	39 (36)	21 (55)	8 (53)
Surgery, n (%)	57 (34)**	13 (8)**	40 (33)	8 (30)	9 (50)	6 (6)	6 (16)	1 (7)
Intolerances/allergies, n (%)	62 (37)	44 (27)	40 (33)	13 (48)	9 (50)	23 (21)^a^	16 (42)^b^	5 (33)^a, b^

Data are presented as mean ± SD for normally distributed data. Categorical data are presented as n (%). * *p* < 0.05 ** *p* < 0.001

Abbreviations: *CD* Crohn’s disease, *UC* Ulcerative colitis, *BMI* Body mass index^a, b^groups with the same superscript letters do not differ significantly after post-hoc analyses using the Bonferroni test (*p* > 0.05)

^c^Education level: no education, primary or lower vocational education and lower general secondary education (low); secondary vocational education and higher general secondary education (middle); higher vocational education and university (high)

### Dietary intake and inflammatory potential

**Fig. 2:**
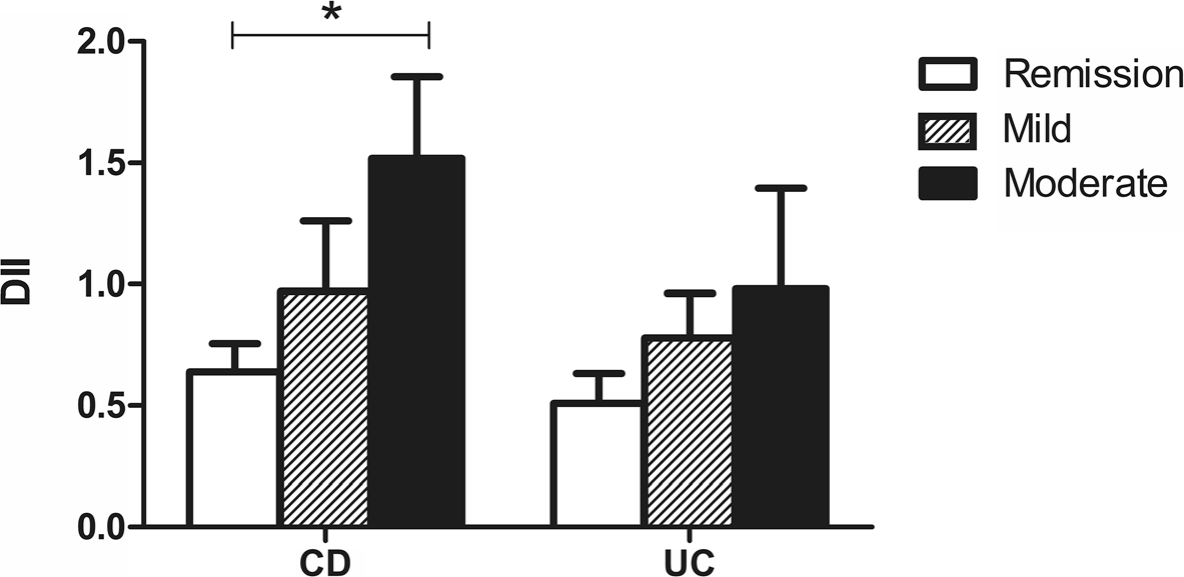
Mean DII of CD (*n* = 168) and UC (*n* = 161) participants stratified for disease activity Abbreviations: CD: Crohn’s disease, UC: ulcerative colitis, DII: dietary inflammatory index. Error bars represent standard error. * indicates significant difference between groups (*p* = 0.03)

The total energy intake and intakes of protein, carbohydrates and fat were not significantly different between CD and UC. In the total population, the DII ranged from − 2.32 to 4.10 with a mean of 0.71 ± 1.33, suggesting a slightly pro-inflammatory diet. No significant differences were found between CD and UC (0.79 ± 1.37 vs 0.62 ± 1.28, *p* = 0.245) (Table 2). Multiple linear regression showed a positive association for CD disease activity scores and the DII in the crude model (β = 12.96; *p* = 0.002), suggesting that CD participants with a higher disease activity consumed a more pro-inflammatory diet. After adjustment for age, age at diagnosis, gender, BMI and education level, this association remained (β = 11.86; *p* = 0.008). No significant association was found between UC disease activity scores and the DII (*p* = 0.307) (Table 3). Across CD and UC disease activity groups, no significant differences were found regarding total energy, protein, carbohydrates and fat intake. In CD, the mean DII was significantly more pro-inflammatory when disease activity was higher (*p* = 0.027), with a significant difference between the remission and moderately active disease group after post-hoc analyses (*p* = 0.030) (Table 2*/*Fig. 2). The parameters that contributed to differences in the DII were alcohol, mono- and polyunsaturated fatty acids, n-3 and n-6 fatty acids, magnesium, selenium, vitamin A, vitamin D and niacin, all having anti-inflammatory effect scores. Lower intakes of these parameters led to a less anti-inflammatory, so a more pro-inflammatory diet, in participants with a higher disease activity.

**Table 2:** Disease activity and inflammatory potential of diet of CD and UC participants and stratified for disease activity

			Disease activity CD			Disease activity UC		
	CD	UC	Remission	Mild	Moderate	Remission	Mild	Moderate
**Disease activity**								
sCDAI score	93 [47–156]	–	79 [44–103]^a^	171 [165–191]^b^	269 [233–326]^c^	–	–	–
Range	44–357	–	44–146	150–218	220–357	–	–	–
P-SCCAI score	–	1 [1–3]	–	–	–	1 [1–1]^a^	3 [3–4]^b^	7 [6–9]^c^
Range	–	0–13	–	–	–	0–2	3–5	6–13
**Dietary intake**								
Nutrient intake								
Energy (kcal)	1912 ± 618	2011 ± 565	1958 ± 601	1789 ± 602	1780 ± 738	2050 ± 587	1919 ± 496	1958 ± 577
Protein, EN%	15.0 ± 2.5	15.3 ± 2.7	15.1 ± 2.4	15.0 ± 2.2	14.6 ± 3.1	15.2 ± 2.8	15.4 ± 2.6	15.2 ± 2.6
Carbohydrates, EN%	43.4 ± 7.3	42.5 ± 6.4	42.9 ± 7.4	45.6 ± 6.6	43.9 ± 7.2	42.2 ± 6.8	43.1 ± 5.7	43.1 ± 4.6
Fat, EN%	36.4 ± 6.2	36.5 ± 5.5	36.7 ± 6.1	34.4 ± 6.1	37.3 ± 6.8	36.6 ± 5.8	36.1 ± 5.2	37.3 ± 4.4
DII	0.79 ± 1.37	0.62 ± 1.28	0.64 ± 1.29^a^	0.97 ± 1.51^a, b^	1.52 ± 1.42^b^	0.51 ± 1.28	0.78 ± 1.13	0.98 ± 1.61
Range	−2.22 - 3.99	− 2.32 - 4.10	− 2.22 - 3.94	− 1.53 - 3.61	− 1.06 - 3.99	−2.32 - 3.46	−1.04 - 2.64	− 0.86 - 4.10

Data are presented as mean ± SD for normally distributed data or median [interquartile range] when skewed. Categorical data are presented as n (%)

Abbreviations: *CD* Crohn’s disease, *UC* Ulcerative colitis, *sCDAI* Short Crohn’s Disease Activity Index, *P-SCCAI* Patient Simple Clinical Colitis Activity Index, *EN%* Energy percent, *DII* Dietary inflammatory index

^a, b^groups with the same superscript letters do not differ significantly after post-hoc analyses using the Bonferroni test (*p* > 0.05)

**Table 3:** Results of multiple linear regression of the association between DII and disease activity as continuous variables, for CD and UC

		CD (***n*** = 168)		UC (***n*** = 161)	
		β-coefficient (95% CI)	***p***-value	β-coefficient (95% CI)	***p***-value
**DII**	Crude	12.96 (4.74–21.18)	**0.002**	0.145 (− 0.134–0.424)	0.307
	Adjusted*	11.86 (3.14–20.58)	**0.008**	0.062 (− 0.236–0.361)	0.681

Abbreviations: *CD* Crohn’s disease, *UC* Ulcerative colitis, *DII* Dietary inflammatory index, *CI* Confidence interval

CD disease activity scores can range from 0 to > 450 and UC disease activity scores can range from 0 to 19

Bold values are significant

*Adjusted model: adjusted for age (years), age at diagnosis (years), gender (m/f), BMI (kg/m^2^) and education level (low/middle/high)

### Patient reported impact and modification of diet

Of the 329 participants, 216 participants (66%) reported that diet had an impact on the course of their disease (73% CD vs 58% UC; *p* = 0.007). Since their diagnosis, 216 participants (66%) have adjusted their dietary intake. Generally, they reported avoidance of certain food products instead of a higher intake of beneficial foods. Lactose-containing products were mostly mentioned to be avoided (37%) followed by spicy foods (23%). Furthermore, participants reported that they reduced their intake of fat (20%), meat (18%), sugar (12%) and onions (11%). No differences were found between CD and UC or disease activity groups. Of the 329 participants, 194 participants (59%) adjust their diet during a flare-up.

## Discussion

In this study, disease activity was associated with the inflammatory potential of diet in participants with Crohn’s disease. Participants with a more pro-inflammatory diet seem to have a higher disease activity. Whether this association is causal remains unclear. In participants with ulcerative colitis, the association was not significant. The majority of participants reported impact of diet on their disease. Modification of diet since diagnosis and during flare-ups was common in both patient groups and all disease activity groups.

To our knowledge, this is the first study to investigate the association between the inflammatory potential of diet and disease activity in patients with IBD in such a large sample. The DII has been calculated previously in a case control study including 62 UC patients and 124 controls to predict the risk of UC based on the inflammatory potential of diet. In that cross-sectional study, participants with higher DII scores seemed to have a higher risk of UC [21]. An analysis of three large prospective cohorts showed that dietary patterns with high inflammatory potential were associated with an increased risk of developing CD, but not UC, though they used a slightly different inflammatory index [2]. In contrast to our results, a recently published cross-sectional study in Iranian patients with an established IBD diagnosis did not find an association between the inflammatory potential of diet and disease activity [22]. The null finding in the Iranian patients might be due to the small sample size (*n* = 143), especially the small number of participants with CD (*n* = 32), as compared to 178 CD patients in the present study.

The DII consists of 45 food parameters that all have their own inflammatory effect score. The inflammatory effect scores we used varied from − 0.663 for fibre to + 0.373 for saturated fat. The contribution of a food parameter to the total DII score depends on the individuals’ intake and the deviation of the inflammatory effect score from 0. Of the food parameters available in this study, fibre, vitamin A, B6, C, D, E, magnesium, zinc, poly-unsaturated fatty acids and n-3 fatty acids had the largest anti-inflammatory impact. Total fat and saturated fat had the largest pro-inflammatory impact. These anti- and pro-inflammatory effects are all based on general inflammation, but seem to be in line with IBD specific nutrient studies. The anti-inflammatory potential of fibre is in line with a prospective study in which they found that high consumption of dietary fibre reduces the risk of relapse among CD patients [23]. By bacterial fermentation of dietary fibre, the production of short-chain fatty acids increases which has anti-inflammatory effects [24]. However, in both the abovementioned study and the DII, no distinction is made between the fermentability, solubility and viscosity of fibres, factors that influence the therapeutic effects of consumption [25]. Regarding the before mentioned vitamins and minerals, several reviews have described the effect of deficiencies on IBD [26–28]. For example, clinical disease activity increases and quality of life decreases significantly with lower levels of vitamin D, and zinc deficiency was shown to be correlated with inflammation in IBD by increasing the number of pro-inflammatory cells [26–28]. Poly-unsaturated and especially n-3 fatty acids have mostly been investigated in the context of supplementation with controversial results [29]. Beneficial effects have been shown, although a clear protective effect in preventing clinical relapse is not demonstrated [29]. A prospective study in patients with CD showed that a diet higher in total fat, saturated fat and a higher ratio of n-6:n-3 fatty acids was associated with disease relapses, which is in line with the inflammatory effect scores of the DII [30]. It seems reasonable to increase dietary intake of n-3 fatty acids for anti-inflammatory effects taking into account the involvement of n-3 fatty acids in immunological and inflammatory responses and an imbalance in n-6:n-3 fatty acid ratio to be a powerful pro-inflammatory stimulus [31]. All before mentioned studies combined, it is likely that the DII reflects influence of diet not only on general inflammation, but also on inflammation in IBD.

The differences in associations of inflammatory potential of diet with disease activity between CD and UC found in this study, are in line with previous studies. Regarding dietary intake, trends are observed in CD as well as in UC, but significant effects are more commonly found in CD [32]. Although we had to use a different disease activity questionnaire for each type of IBD, there is a similar distribution of disease activity groups in CD and UC. This distribution makes it unlikely that the different questionnaires account for the differences in associations between CD and UC. We did not assess the affected part of the gastrointestinal tract. Therefore, it is not possible to correct the associations for this aspect, although the affected part of the gastrointestinal tract may explain differences in associations between CD and UC.

Dietary beliefs of the participants in our study are in line with previous surveys. A similar percentage (58–62%) reported that diet influences their disease course and that avoidance of certain food products is preferred over a higher intake of beneficial foods [5–7].

Strengths of this study include the large number of participants, which enabled us to perform analyses for CD and UC separately, and the use of validated questionnaires to determine disease activity and dietary intake. However, some limitations should be mentioned. This was a cross-sectional study and any association could therefore be a result of reverse causality. Participants who experience more pain or discomfort because of their disease may have changed their diet to relieve symptoms. Besides that, our sample included mainly participants in remission. A sample with an equal number of participants in each disease activity category would have increased the power to find stronger associations. Next to this, some extent of bias possibly occurred. More women than men responded, and the education level in our sample was high. In a large German study that compared responders to non-responders, women (up to 50 years old) were more likely to respond than men, as were non-smokers and those with a high education level [33]. As diet quality is related to gender, education level, and various other lifestyle factors [34], this could mean that on average, our study sample had a more healthy diet than that of the average IBD patient, corresponding with a lower DII (more anti-inflammatory). Although, the range in DII was wide enough to find associations with disease activity, putting more effort into recruiting patients with more active disease or an unhealthy diet would be recommendable for the future. However, we do not think that selection bias negatively affected our study results, Another limitation is that we could not perform the complete calculation of the DII, because not all food parameters could be assessed with the FFQ or were not available in the food composition database. Those food parameters, mainly flavonoids, herbs and spices, all had anti-inflammatory effect scores. Therefore, our results were probably more directed towards pro-inflammatory scores. Using less food parameters generally also results in a lower variation in DII [11]. However, all other studies that calculated the DII also were unable to include all 45 food parameters and used a number of food parameters that was comparable to our study [12–14, 20, 21]. The DII only consists of food parameters, supplement use is not included. In our study, more than 40% of the participants used a food supplement, including vitamin supplements. Most vitamins and minerals do have anti-inflammatory effect scores, which might have led to an underestimation of the DII in our study. As we did not have information about brands and dosages of the supplements, it was not possible to incorporate these in the calculation of the DII. However, supplement use was equal in CD and UC participants as well as all disease activity groups, which makes it less likely that supplement use affected the association between DII and disease activity. Finally, for the outcome disease activity, we did not use an objective marker, but based it on a questionnaire, which was filled in by the participant instead of a physician. However, previous studies have validated the sCDAI and P-SCCAI and concluded that both are reliable and feasible for disease activity measurement. For both participant-based disease activity questionnaires, significant correlations were found with the physician-based questionnaires as well as biomarkers such as CRP [15, 16].

## Conclusions

In conclusion, we found an association between the inflammatory potential of diet and disease activity in Crohn’s disease, but not in ulcerative colitis. Although this association does not prove a causal relationship, for daily practice it suggests that a diet high in anti-inflammatory nutrients such as fibre, n-3 fatty acids, vitamins and minerals, which is a diet in line with current nutritional guidelines for healthy adults, seems to be equally prudent in IBD patients, especially Crohn’s disease. Longitudinal studies are needed to further investigate the effect of diet on the course of disease.

## Data Availability

All data generated and/or analysed during the current study are available from the corresponding author on reasonable request.

## Abbreviations

ANOVA: Analysis of variance
BMI: Body mass index
CD: Crohn’s disease
CI: Confidence interval
DII: Dietary Inflammatory Index
EN%: Energy percent
FFQ: Food Frequency Questionnaire
IBD: Inflammatory bowel disease
IQR: Interquartile range
P-SCCAI: Patient Simple Clinical Colitis Activity Index
sCDAI: Short Crohn’s Disease Activity Index
SD: Standard deviation
UC: Ulcerative colitis
